# Whole-Genome Sequencing and Antibiotic Resistance Profiling of *Helicobacter pylori* Isolates from a Tertiary Hospital in Southern Thailand

**DOI:** 10.3390/antibiotics14090944

**Published:** 2025-09-18

**Authors:** Chonticha Romyasamit, Apichat Kaewdech, Pimsiri Sripongpun, Naichaya Chamroonkul, Komwit Surachat, Sirikan Suwannasin, Yosita Leepromma, Morteza Saki, Maseetoh Samaeng, Phoomjai Sornsenee

**Affiliations:** 1 Department of Medical Technology, School of Allied Health Sciences, Walailak University, Nakhon Si Thammarat 80160, Thailand; chonticha.ro@wu.ac.th (C.R.); ninettex0305@gmail.com (Y.L.); 2 Research Center in Tropical Pathobiology, Walailak University, Thasala District, Nakhon Si Thammarat 80160, Thailand; 3 Gastroenterology and Hepatology Unit, Division of Internal Medicine, Faculty of Medicine, Prince of Songkla University, Hat Yai, Songkhla 90110, Thailand; apichat.ka@psu.ac.th (A.K.); spimsiri@medicine.psu.ac.th (P.S.); naichaya@gmail.com (N.C.); 4 Department of Biomedical Sciences and Biomedical Engineering, Faculty of Medicine, Prince of Songkla University, Hat Yai, Songkhla 90110, Thailand; komwit.s@psu.ac.th (K.S.); sirikan4036@gmail.com (S.S.); 5 Department of Microbiology, Faculty of Medicine, Ahvaz Jundishapur University of Medical Sciences, Ahvaz, Iran; mortezasaki1981@gmail.com; 6 Division of Internal Medicine, Faculty of Medicine, Prince of Songkla University, Hat Yai, Songkhla 90110, Thailand; fsamaeng@yahoo.com; 7 Family Medicine and Preventive Medicine, Faculty of Medicine, Prince of Songkla University, Songkhla 90110, Thailand

**Keywords:** *Helicobacter pylori*, whole-genome sequencing, drug resistance, virulence, gastric cancer, Thailand

## Abstract

**Background**: *Helicobacter pylori* is associated with a wide range of gastroduodenal diseases, including chronic gastritis, peptic ulcer disease, and gastric cancer. Eradication efforts are challenged by increasing antimicrobial resistance rates, particularly in Southeast Asia. We sequenced the whole genomes of clinical *H. pylori* isolates from Southern Thailand to elucidate their resistance profiles, virulence determinants, and evolutionary relationships. **Methods**: Three clinical *H. pylori* isolates (004, 117, and 189) were subjected to whole-genome sequencing, phenotypic antimicrobial susceptibility testing, and comparative genomic analyses. **Results**: All strains exhibited high-level resistance to metronidazole. Additionally, *H. pylori* 117 was resistant to both amoxicillin and levofloxacin, classifying it as multidrug-resistant. Genomic analysis revealed mutations in *rdxA*, *frxA*, and *rpoB*, as well as in penicillin-binding protein genes (*pbp2* and *pbp3*), supporting the phenotypic findings. While all isolates harboured clarithromycin resistance mutations (A2142G and A2143G in the 23S rRNA gene), they were phenotypically susceptible, highlighting a potential discordance that requires further investigation. Virulence gene profiling identified 115–118 conserved genes per strain, including *cagA*, *vacA*, *oipA*, *babA*, and flagellar, urease, and lipopolysaccharide biosynthesis genes. Phylogenetic analysis using core-genome single-nucleotide polymorphisms demonstrated that these strains formed a distinct Southern Thai monophyletic clade, suggesting localised clonal expansion driven by regional selective pressures. **Conclusions**: Region-specific surveillance strategies and treatment guidelines are urgently needed in Thailand. The combination of high-risk virulence genes and rising antimicrobial resistance in *H. pylori* strains necessitates tailored therapeutic approaches, the integration of genomic surveillance into clinical diagnostics, and expanded studies linking genotype to clinical outcomes in diverse populations.

## 1. Introduction

*Helicobacter pylori* is a Gram-negative, spiral-shaped, flagellated, microaerophilic bacterium that colonises the human gastric mucosa. Its unique morphology and urease activity enable it to survive in the acidic gastric environment by facilitating the penetration of the mucus layer and neutralisation of local acidity, leading to chronic inflammation and disruption of epithelial homeostasis [1,2]. *H. pylori* has been implicated in the development of several upper gastrointestinal diseases, including chronic gastritis, peptic ulcer disease, and gastric mucosa-associated lymphoid tissue lymphoma [3,4].

The International Agency for Research on Cancer has designated *H. pylori* as a Group 1 carcinogen because of its established role in gastric cancer [5]. *H. pylori* has been suggested to be associated with extraintestinal diseases, such as immune thrombocytopenic purpura, refractory iron deficiency anaemia, and vitamin B12 deficiency. Around half of the global population is estimated to harbour this bacterium, although prevalence rates differ markedly by region. Higher infection rates are consistently reported in low- and middle-income countries, particularly in parts of Asia, Africa, and Latin America, where factors such as limited sanitation infrastructure, lower socioeconomic status, and household crowding contribute to the transmission of the disease [6]. The primary routes of *H. pylori* dissemination are believed to be the faecal–oral and oral–oral routes. According to a comprehensive systematic review and meta-analysis encompassing 224 studies conducted in 71 countries, the global prevalence of *H. pylori* infection between 2011 and 2022 was estimated at 43.1% [7]. In Thailand, the seroprevalence of *H. pylori* is estimated to be 45–60%, with some studies suggesting regional variations. Despite the moderate infection rate, gastric cancer remains a significant cause of cancer-related mortality in the country. Southern Thailand has been recognised as a key surveillance area due to its high antibiotic usage combined with unique dietary patterns [8,9].

The pathogenicity of *H. pylori* largely depends on an array of virulence factors that modulate host interactions and influence disease severity. The *cagA* gene encodes a cytotoxin-associated protein that is translocated via a type IV secretion system into gastric epithelial cells, where it interferes with intracellular signalling cascades and contributes to oncogenic processes [10]. The *vacA* gene encodes a vacuolating cytotoxin implicated in epithelial injury and evasion of the host immune response [10,11]. Adhesin genes, including *babA*, *oipA*, and *sabA*, facilitate bacterial attachment to the gastric mucosa and promote inflammatory responses, thereby enhancing pathogenic capacity [10]. The distribution and expression of these virulence determinants vary among strains and are associated with geographic variation in disease manifestations, particularly the risk of gastric cancer [12].

Eradication of *H. pylori* infection typically relies on combination regimens that include a proton pump inhibitor (PPI) in conjunction with two or more antibiotics. Common first-line treatments include clarithromycin-based triple therapy, consisting of a PPI, clarithromycin, and either amoxicillin or metronidazole, and bismuth quadruple therapy, which uses a PPI along with bismuth, tetracycline, and metronidazole [12]. However, antimicrobial resistance (AMR) is increasing worldwide and is a primary factor contributing to the failure of *H. pylori* eradication therapies. Resistance is predominantly caused by chromosomal point mutations rather than horizontal gene transfer [12].

Clarithromycin resistance, which has a considerable clinical impact, is primarily linked to point mutations A2142G and A2143G within the 23S rRNA gene, disrupting antibiotic binding at the ribosomal site [10]. Metronidazole resistance often results from loss-of-function mutations in *rdxA* and *frxA*, which encode nitroreductase enzymes required for prodrug activation [10]. While resistance to amoxicillin and tetracycline is still relatively uncommon worldwide, mutations in *pbp1A* and the 16S rRNA gene are emerging and may compromise therapeutic efficacy [10]. In Thailand, several studies have reported clarithromycin resistance rates ranging from 18% to over 30%, whereas the metronidazole resistance rate frequently exceeds 50%, particularly in southern provinces [8,9]. The increasing prevalence of resistant strains has contributed to reduced treatment success and underscores the need for bismuth-based quadruple regimens or individualised therapies guided by susceptibility testing.

The contribution of virulent or resistant *H. pylori* strains to this burden remains poorly characterised, particularly in Southern Thailand, where genomic surveillance is limited. While phenotypic assessments provide important insights, they do not reveal the underlying genetic mechanisms of resistance and pathogenicity. Genomic approaches are thus essential for uncovering mutations, tracking strain evolution, and identifying potential markers for clinical outcomes. This study aimed to comprehensively characterise three clinical *H. pylori* isolates from a tertiary hospital in Southern Thailand through an integrated phenotypic and genomic approach. We analysed AMR patterns, identified resistance-associated mutations, profiled virulence gene distribution, constructed phylogenetic trees, and evaluated associations with gastric cancer. We expected our findings to enhance the understanding of regional resistance dynamics, support rational treatment selection, and contribute to the development of gastric cancer prevention strategies.

## 2. Results

### 2.1. Isolation and Identification of H. pylori

**Figure 1:**
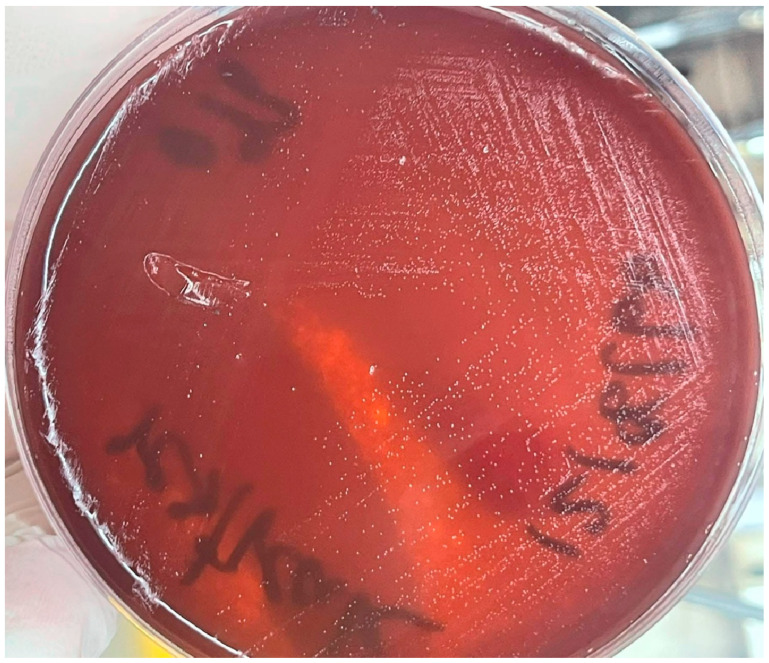
*H. pylori* growth on Columbia agar base supplemented with 7% horse blood.

Three *H. pylori* isolates were successfully recovered from gastric biopsy specimens. Based on culture and microbiology, they were identified as *H. pylori*, and matrix-assisted laser desorption ionisation-time-of-flight mass spectrometry (MALDI-TOF MS) log (score) values were >2.0 for all isolates, indicating reliable species-level identification. Colonies were typically small, circular, grey in colour, and translucent (Figure 1).

### 2.2. Antimicrobial Susceptibility Profiles

Antimicrobial susceptibility profiles of the isolates are summarised in Table 1. All isolates demonstrated high-level resistance to metronidazole (MIC > 256 µg/mL). One isolate (*H. pylori* 117) exhibited multidrug resistance, including resistance to amoxicillin and levofloxacin. In contrast, all isolates were susceptible to clarithromycin, with MICs ranging from 0.032 to 0.094 µg/mL.

### 2.3. Genomic Features of the H. pylori Isolates

**Figure 2:**
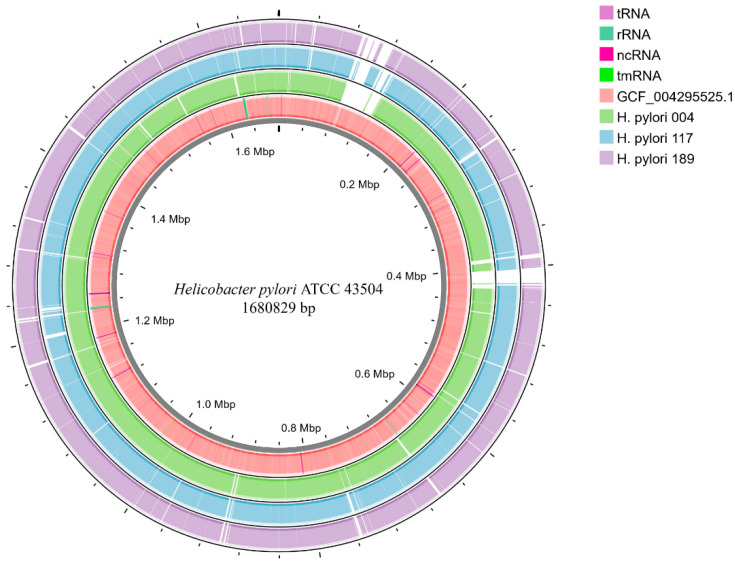
Comparative genome-wide analysis of *H. pylori* strains 004, 117, 189, and ATCC 43504.

The overall genomic characteristics of the *H. pylori* isolates revealed notable differences in key features. Draft genome assemblies of strains 004, 117, and 189 had total sizes ranging from 1,559,464 to 1,595,107 bp (Table 2 and Figure 2). Specifically, the genome of *H. pylori* 004 comprised 29 contigs, totalling 1,587,334 bp, encoding 1587 coding sequences (CDSs), 36 transfer (t)RNAs, two ribosomal (r)RNA operons, and one transfer-messenger (tm)RNA, with a GC content of 38.9%. *H. pylori* 117 consisted of 25 contigs spanning 1,559,464 bp, comprising 1555 CDSs, 36 tRNAs, and two rRNAs, with a GC content of 38.9% (the same as that of strain 004). The *H. pylori* 189 assembly included 32 contigs, totalling 1,595,107 bp, with 1625 CDSs, 36 tRNAs, two rRNAs, and a GC content of 39.1%. Average nucleotide identity analysis confirmed the species assignment to H. pylori, with all isolates exhibiting greater than 95% identity to the reference genome (Table 2). The reference genome of *H. pylori* strain ATCC 43504 consisted of two assembled contigs, with a combined genome size of approximately 1.6 Mbp (Figure 2).

### 2.4. Comparative Genomics

Comparative genomic analyses were performed to characterise similarities and differences among the three sequenced *H. pylori* isolates and selected reference genomes, including ATCC 43504. Circular genome visualisations were generated using the BLAST+ 2.16.0 Ring Image Generator and annotated with Proksee (Figure 2). Alignment of the draft assemblies revealed high overall sequence similarity (>98%) between each isolate and the ATCC 43504 reference genome. Multiple regions exhibited putative insertions, deletions, or sequence divergence, reflecting the characteristic genomic plasticity of *H. pylori*. Annotation confirmed that all isolates encoded a comparable number of CDSs, tRNAs, and rRNAs, consistent with prior reports of the *H. pylori* core genome. Visualisation of the GC content and CDS distribution further supported their classification within the species, as corroborated by average nucleotide identity analyses (Table 2 and Figure 2).

### 2.5. Pan-Genome Analysis and Phylogenetic Relationships

**Figure 3:**
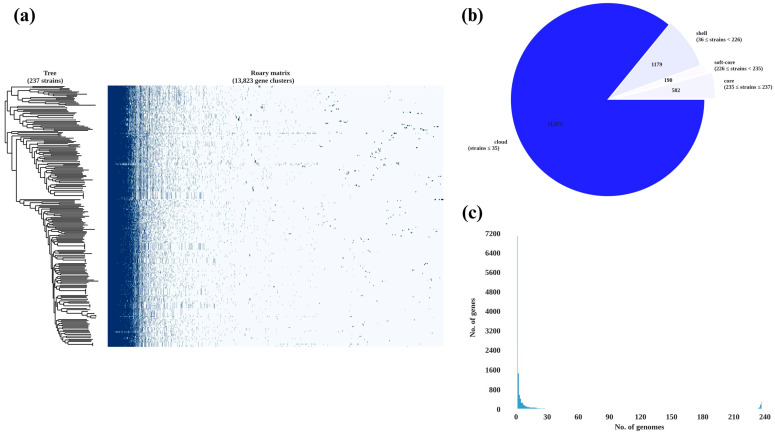
Pan-genome analysis of *H. pylori* 237 isolates. (**a**) presence/absence matrix; (**b**) Pie chart of gene categories; (**c**) Gene frequency histogram.

**Figure 4:**
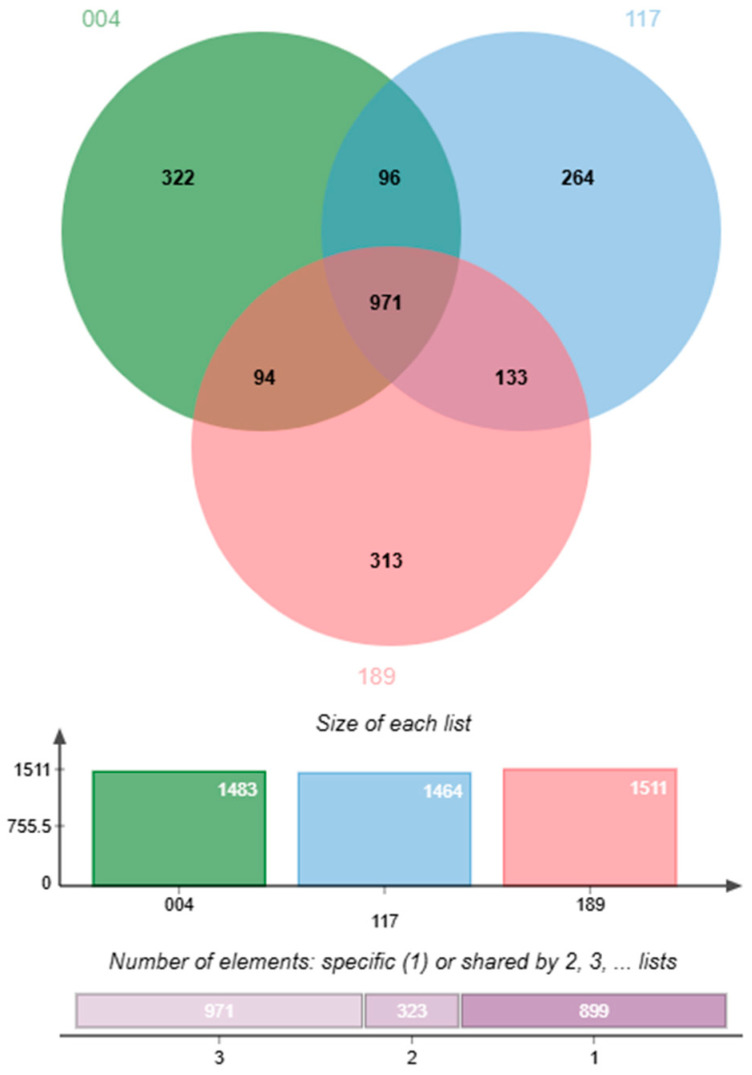
Venn diagram of genes unique to and shared among *H. pylori* isolates 004, 117, and 189, generated using jvenn.

A pan-genome analysis of the three *H. pylori* isolates and 234 publicly available *H. pylori* genomes identified a conserved core genome as well as a substantial set of accessory genes (Figure 3). The presence—absence matrix revealed a high proportion of shared genes. However, each isolate also retained a distinct subset of unique gene clusters, underscoring the genomic diversity within Thai clinical strains. A detailed comparison of the three Thai isolates showed that they shared 971 gene clusters, representing the core genome. Unique gene clusters were also identified in each isolate: 322 in *H. pylori* 004, 264 in *H. pylori* 117, and 313 in *H. pylori* 189. Pairwise comparisons identified 96 gene clusters shared exclusively between *H. pylori* 004 and *H. pylori* 117, 94 between *H. pylori* 004 and *H. pylori* 189, and 133 between *H. pylori* 117 and *H. pylori* 189. In total, 1483 gene clusters were detected in *H. pylori* 004, 1464 in *H. pylori* 117, and 1511 in *H. pylori* 189. These findings categorise the pan-genome into three main components: a core genome (971 genes), an accessory genome shared between pairs (323 genes), and a unique genome set specific to individual strains (899 genes), as visualised in the Venn diagram in Figure 4.

**Figure 5:**
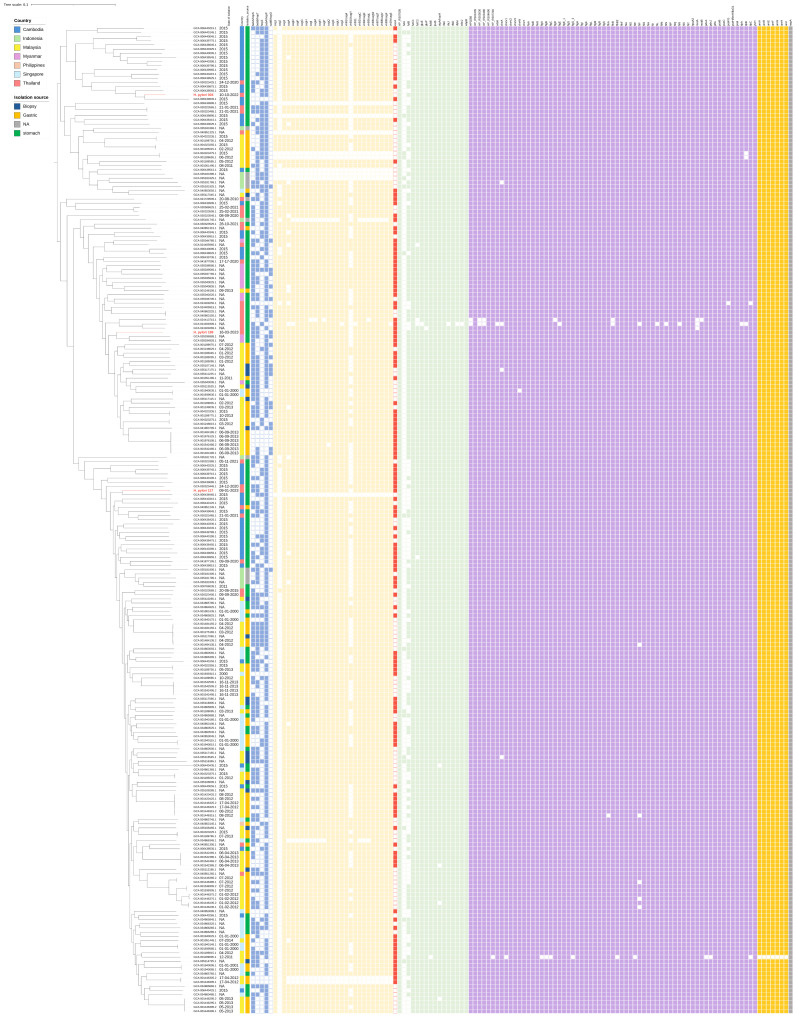
Phylogenetic analysis of 237 *H. pylori* strains based on core genome single-nucleotide polymorphisms.

To investigate evolutionary relationships, a maximum-likelihood phylogenetic tree was constructed based on core single-nucleotide polymorphisms (Figure 5). The three Thai isolates clustered closely together, forming a distinct monophyletic clade that was separate from the Southeast Asian reference strains included in the analysis. These findings suggest substantial genomic diversity among Thai isolates, with potential implications for the evolution of antibiotic resistance and local treatment strategies.

### 2.6. AMR Gene Profiling

Whole-genome sequencing of strains 004, 117, and 189 revealed multiple AMR determinants (Table 3). All three isolates carried point mutations A2143G and A2142G in the 23S rRNA gene, which are commonly associated with clarithromycin resistance. Mutations in *rdxA* and *frxA*, genes involved in metronidazole activation, were identified in each strain. Additionally, substitutions within the quinolone resistance-determining regions of *gyrA* and *gyrB* were detected, indicating potential fluoroquinolone resistance. The *pbp2* gene exhibited mutations in all isolates, whereas *pbp3* mutations were specific to strain 117. Analysis using the Resistance Gene Identifier tool predicted the presence of additional AMR-associated genes: *vanT* (*vanG* cluster) in strains 004 and 189; *vanTr* (*vanL* cluster) in strain 117; and *rpoB* in strain 004.

### 2.7. Virulence Gene Profiling

Whole-genome analysis of the three strains identified 115 to 118 virulence-associated genes per strain (Table S1). All three isolates carried major pathogenicity-related genes, including *cagA*, *cagL*, and other genes encoding components of the type IV secretion system, *vacA*, *oipA*, and *babA/hopS*. Genes involved in flagellar assembly (e.g., *flaA*, *flgE*, and *fliG*), chemotaxis (*cheA* and *cheY*), urease activity (*urea* and *ureB*), and lipopolysaccharide biosynthesis (*lpxB*, *rfaC*, and *wbpB*) were consistently detected across all strains and exhibited high sequence similarity.

Strain-specific differences were observed in genes encoding several outer membrane proteins. For example, *hopZ* was present in strain 004 but absent in strains 117 and 189, whereas *sabB/hopO* was identified only in strain 189. Pairwise sequence identities of the virulence genes ranged from 86.74% to 98.88%, indicating high conservation among functional categories, including adherence, motility, immune modulation, and toxin secretion.

## 3. Discussion

Three clinical isolates, *H. pylori* 004, 117, and 189, were successfully isolated and analysed. All strains showed resistance to metronidazole, and strain 117 was classified as multidrug-resistant. Whole-genome analysis confirmed that all three isolates shared a well-conserved group of virulence genes, suggesting a high potential for disease development. The isolates carried a concerning combination of strong virulence factors, widespread AMR, and a distinct genetic background.

The high prevalence of metronidazole resistance observed in our isolates aligns with the increasing rates reported across developing countries, where resistance rates often exceed 50%, in contrast to the 20–40% typically reported in Western populations [13,14]. In a previous study in a Thai cohort of 291 individuals, *H. pylori* infection was identified in 51.2% of participants [8]. Among those infected, AMR was detected in 75.8% of isolates, with notably high resistance rates to metronidazole (71.8%) and levofloxacin (19.4%). Notably, multidrug resistance, defined as resistance to at least two different classes of antibiotics, was observed in 21.8% of the isolates [8]. Previous studies have shown that mechanisms underlying metronidazole resistance in *H. pylori* may involve decreased intracellular drug uptake, enhanced bacterial oxidative stress response, improved DNA repair pathways, and the detoxification of metronidazole-derived reactive intermediates [15]. Genomic analysis of the three *H. pylori* isolates revealed mutations in *rdxA* and *frxA*, both of which encode oxygen-insensitive nitroreductases essential for the activation of metronidazole. Disruptions in these genes are known to impair the enzymatic reduction in the prodrug, leading to decreased susceptibility and high-level resistance. Although *fdxB* mutations have also been associated with resistance, alterations in *rdxA* and *frxA* remain the most consistently reported mechanisms contributing to metronidazole resistance in *H. pylori* [16,17]. The diversity of the mutations observed suggests multiple independent evolutionary events rather than clonal spread of a single resistant lineage [16,17].

*H. pylori* 004 and 117 harboured mutations in *pbp2* and *pbp3*, encoding penicillin-binding proteins involved in cell wall synthesis. These findings align with established mechanisms of amoxicillin resistance in *H. pylori*, which are primarily attributed to alterations in drug-binding affinity-reducing properties of PBPs, particularly in PBP1 [18,19]. The finding suggests a broader mutational spectrum contributing to reduced susceptibility. It underscores a potentially evolving resistance mechanism, challenging the long-standing assumption of amoxicillin’s uniformly high efficacy in *H. pylori* eradication therapy.

A mutation in *rpoB* was detected in *H. pylori* strain 004. The *rpoB* gene encodes the β-subunit of RNA polymerase, which is the target of rifamycin antibiotics. Mutations in *rpoB* have been associated with resistance to rifampin and, in some cases, to fluoroquinolones such as levofloxacin. This cross-resistance may result from altered transcriptional regulation or compensatory mechanisms that promote bacterial survival under antimicrobial pressure. The detection of an *rpoB* mutation in a clinical isolate from Southern Thailand highlights the growing complexity of antimicrobial resistance in *H. pylori*, particularly in areas with high antibiotic use. Interestingly, a notable discordance was observed between genotypic and phenotypic resistance to clarithromycin. Although all three isolates had resistance-associated mutations in the 23S rRNA gene (A2143G and A2142G), they were phenotypically susceptible to clarithromycin. Similar inconsistencies have been reported for other bacterial species as well. They may result from complex regulatory factors, such as gene expression modulation, gene dosage effects, or compensatory mutations that attenuate the resistance phenotype despite the presence of resistance-associated alleles [11,20,21].

Phylogenetic analysis showed that the Thai *H. pylori* isolates formed a distinct monophyletic clade separate from other Southeast Asian strains. This finding supports the hypothesis of region-specific evolutionary adaptation and suggests local clonal expansion. Such biogeographic structuring aligns with global patterns observed in *H. pylori* populations, where bacterial phylogeny often mirrors historical human migrations and demographic transitions [22,23]. The genetic separation of the Thai strains from nearby geographic populations may reflect localised evolutionary pressures, including founder effects, geographic isolation, and long-term host–pathogen coevolution. These insights have epidemiological significance, highlighting that antimicrobial resistance profiles and virulence factor repertoires may vary substantially even across geographically proximate populations, necessitating region-specific surveillance and therapeutic strategies [22]. The substantial genomic diversity observed among the three closely related isolates, with each harbouring between 264 and 322 unique genes, exemplifies the remarkable evolutionary flexibility that has enabled *H. pylori* to persist as a successful human pathogen for millennia. This genomic plasticity is mediated by high mutation rates, frequent recombination events, and occasional horizontal gene transfer, which collectively contribute to the species’ ability to adapt to diverse host environments and evade immune responses [24,25]. The presence of strain-specific variations in outer membrane protein genes likely reflects an ongoing adaptive evolution in response to host immune pressures and environmental challenges, as these proteins play crucial roles in bacterial adherence, immune evasion, and nutrient acquisition [26,27].

Our genomic characterisation of clinical *H. pylori* isolates from Southern Thailand provides a comprehensive view of their virulence potential, resistance mechanisms, and evolutionary adaptation, with implications for both clinical management and public health.

Virulome profiling revealed a conserved set of 115–118 virulence-associated genes per strain, including canonical factors such as *cagA*, *vacA*, *babA*, and *oipA*, as well as complete components of the type IV secretion system. The consistent presence of the cag pathogenicity island, which facilitates *CagA* translocation into host epithelial cells, underpins the high pathogenic potential of these isolates [11,28]. Given its association with peptic ulcer disease and gastric cancer, the ubiquitous detection of *cagA* is clinically significant. The *vacA* gene product is responsible for cellular vacuolation [29,30]. Although allele subtypes were not identified, the co-occurrence of *cagA* and *vacA* in all strains suggests synergistic effects on pathogenesis, consistent with findings from other high-burden regions. The consistent presence of *cagA* and *vacA* in the Thai isolates aligns with epidemiological data showing elevated gastric cancer rates in Southeast Asia compared to Western populations and supports the continued clinical significance of *H. pylori* infection in this region [28,31,32].

In Southern Thailand, the widely used triple therapy (PPI + amoxicillin + metronidazole) may no longer achieve acceptable eradication rates due to the uniform, high-level metronidazole resistance observed (all three isolates had MICs > 256 µg/mL) and the emergence of amoxicillin resistance in two strains. Bismuth quadruple therapy (PPI + bismuth + tetracycline + metronidazole) or non-bismuth concomitant regimens (PPI + amoxicillin + clarithromycin + tetracycline) should be used instead, as these can overcome metronidazole resistance when at least two antibiotics are still active. Furthermore, using tetracycline instead of amoxicillin in first-line protocols may further increase efficacy given the pbp2-mediated amoxicillin resistance signals. Tailored regimens directed by rapid PCR panel tests (e.g., 23S rRNA, rdxA/frxA, and pbp2) would improve individual patient outcomes and halt *H. pylori* spread in situations where culture and susceptibility testing are available.

This study is limited by its small sample size, which may not fully represent the diversity of *H. pylori* strains in Southern Thailand. Because all isolates were recovered via culture, selection bias may have excluded fastidious or low-abundance strains. The lack of clinical data limited our ability to correlate genomic features with disease outcomes. Future studies with larger, more diverse samples and clinical metadata are needed to strengthen the findings. Nonetheless, the genomic approach used here provides a valuable model for resistance surveillance in resource-limited settings. The insights from this study are relevant not only for Thailand but also for other Southeast Asian countries facing similar challenges with *H. pylori* prevalence and antimicrobial resistance.

## 4. Materials and Methods

### 4.1. Ethics Statement

This study was approved by the Ethics Committee of the Faculty of Medicine, Prince of Songkla University (REC. 64-409-09-1). Written informed consent was obtained from all participants prior to sample collection, in accordance with the Declaration of Helsinki and institutional guidelines.

### 4.2. Tissue Biopsy

This study was performed according to Vilaichone RK et al., 2016 [8]. Gastric mucosal biopsy specimens were collected from the antrum and corpus of patients undergoing routine upper gastrointestinal endoscopy using standard biopsy forceps. Each specimen was subjected to a rapid urease test (CLO kit, Pronto Dry, Gastrex, Brignais, France; distributed by Medical Instruments Corporation GmbH, Herford, Germany) and incubated at 25 °C. A positive result was defined as a colour change from yellow to pink or red within 24 h, indicating the presence of *H. pylori*.

### 4.3. H. pylori Isolation and Culture

Bacterial isolations were performed according to Vilaichone RK et al., 2016 [8]. Biopsy tissues were inoculated onto Columbia agar plates (Oxoid, Hampshire, UK) supplemented with 7% horse blood at 37 °C in microaerophilic conditions (5% O_2_, 10% CO_2_, and 85% N_2_) for five days. Suspected colonies were preserved at −80 °C in glycerol-containing brain heart infusion broth (HiMedia, Mumbai, India) supplemented with 7% heat-inactivated foetal bovine serum until analysis.

### 4.4. MALDI-TOF MS Identification

Suspected colonies were confirmed using a MALDI-TOF MS system (Bruker Daltonics, Bremen, Germany). Proteins were extracted from all isolates following the manufacturer’s standard protocol, and spectral data were matched against the Bruker reference database. Isolates with log (score) values ≥2.0 were considered reliably identified as *H. pylori*.

### 4.5. Antibiotic Susceptibility Testing

Antimicrobial susceptibility of the confirmed isolates was evaluated using the E-test method (bioMérieux, Marcy-l’Étoile, France). In brief, *H. pylori* cultures were adjusted to a turbidity equivalent to the McFarland standard 3 [33]. E-test strips were placed onto Mueller–Hinton agar (HiMedia, Mumbai, India) supplemented with 5% sheep blood. The following antibiotics were tested: levofloxacin (0.002–32 µg/mL), amoxicillin (0.016–256 µg/mL), clarithromycin (0.016–256 µg/mL), and metronidazole (0.016–256 µg/mL). The plates were incubated at 37 °C under microaerophilic conditions for 72 h. Minimum inhibitory concentrations (MICs) were interpreted according to the 2025 European Committee on Antimicrobial Susceptibility Testing guidelines [34].

### 4.6. Genomic Analysis

#### 4.6.1. DNA Extraction and Whole-Genome Sequencing

Genomic DNA was extracted from three *H. pylori* isolates using a DNeasy extraction kit (Qiagen, Hilden, Germany) per the manufacturer’s instructions. In brief, bacterial cells were initially lysed in 180 µL of lysis buffer at 37 °C for 30 min, followed by the addition of 25 µL of proteinase K and 200 µL of buffer AL and further incubation at 56 °C for 30 min. Subsequently, 200 µL of ethanol was added, and the mixture was subjected to centrifugation, followed by sequential washing with buffer AW2 and final elution in buffer AE. DNA purity was evaluated spectrophotometrically by measuring the A260/A280 ratio and verified by agarose gel electrophoresis.

The purified DNA was submitted to the Beijing Genomics Institute for whole-genome sequencing on the MGISEQ-2000 platform, producing 150 bp paired-end reads. The short-read sequencing data were analysed using the Bactopia v3.0.1 automated pipeline [35]. Genome assembly was performed using Shovill v1.1.0, whereas annotation was carried out using Prokka v1.5.0 [36]. Species identification was conducted based on the Genome Taxonomy Database [37]. Draft genome assemblies were visualised using the Proksee platform [38] against *H. pylori* ATCC 43504 (NZ_AP017632.1). Virulence factors and AMR genes were predicted using the Resistance Gene Identifier with default parameters based on the Virulence Factors Database [39] and the Comprehensive Antibiotic Resistance Database [40]. Mobile genetic elements and prophages were assessed using mobileOG-db (beatrix-1.6) [41] and Phigaro v2.4.0 [42], respectively.

#### 4.6.2. Pan-Genome Analysis and Comparative Genomics

Genome assemblies of 234 *H. pylori* strains from Southeast Asia were retrieved from GenBank on 2 July 2025 (https://www.ncbi.nlm.nih.gov/), as summarised in Table S1. For quality control of genome assemblies, only draft genomes with a sequencing coverage depth greater than 100× were included. Assemblies were further assessed based on N50 values, with a cut-off of ≥20,000 bp, and total genome sizes between 1.6 and 1.7 Mb were considered acceptable. Contigs shorter than 500 bp were excluded. Pan-genome analysis of the three *H. pylori* isolates was conducted using the Roary pipeline [43], using a 95% BLASTp identity threshold and default parameters to categorise core, accessory, and unique protein families. Core single-nucleotide polymorphisms were extracted, and a maximum-likelihood phylogenetic tree was constructed using FastTree v2.1.0 [44]. The resulting tree was visualised with the Interactive Tree of Life v7.0 platform [45]. Venn diagrams depicting genes unique to and shared among *H. pylori* isolates 004, 117, and 189 were generated using the jvenn web tool [46]. Circular genome visualisations were generated using the BLAST Ring Image Generator and annotated with Proksee.

#### 4.6.3. Analysis of Gene Correlations with Gastric Cancer

To explore potential associations between bacterial genes and clinical outcomes, particularly gastric cancer, the presence of known virulence genes (*cagA*, *vacA*, *babA*, and *oipA*) and resistance mutations were compared between isolates from patients with and without gastric cancer.

## 5. Conclusions

This genomic analysis of *H. pylori* isolates from Southern Thailand highlights the emergence of multidrug resistance, including to amoxicillin, posing a significant clinical challenge. The phylogenetic uniqueness and virulence potential of these strains underscore the need for region-specific surveillance and tailored treatment strategies. The study findings establish a framework for future research and emphasise the importance of ongoing genomic monitoring, expanded sampling, and integration of clinical data to effectively address *H. pylori* antibiotic resistance in Thailand and comparable settings.

## Figures and Tables

**Table 1:** *H. pylori* isolate E-test MIC values for amoxicillin, levofloxacin, metronidazole, and clarithromycin.

Isolate	MIC (μg/mL)			
	AMX	LVX	MTZ	CLR
*H. pylori* 004	0.75 (R)	>32 (R)	>256 (R)	0.094 (S)
*H. pylori* 117	>256 (R)	0.19 (S)	>256 (R)	0.032 (S)
*H. pylori* 189	0.023 (S)	1 (S)	>256 (R)	0.047 (S)

The MIC breakpoint was determined using European Committee on Antimicrobial Susceptibility Testing version 12.0. Abbreviations: E-test, Epsilometer test; MIC, minimum inhibitory concentration; AMX, amoxicillin; LVX, levofloxacin; MTZ, metronidazole; CLR, clarithromycin; R, resistant; S, susceptible. MIC Breakpoint: AMX > 0.125 μg/mL; LVX > 1 μg/mL; MTZ > 8 μg/mL; CLR > 0.5 μg/mL.

**Table 2:** Genome characteristics of the *H. pylori* strains.

Feature	*H. pylori* 004	*H. pylori* 117	*H. pylori* 189
Bases (bp)	1,587,334	1,559,464	1,595,107
Contigs (N)	29	25	32
L50 (N)	6	4	7
N50 (N)	82,149	136,963	108,320
GC content (%)	38.9	38.9	39.1
CDSs (N)	1587	1555	1625
rRNAs (N)	2	2	2
tRNAs (N)	36	36	36
tmRNAs (N)	1	1	1
Classification	*H. pylori*	*H. pylori*	*H. pylori*
FastANI_reference	GCF_009689985.1	GCF_000277405.1	GCF_000277405.1

**Table 3:** Comprehensive Antibiotic Resistance Database Resistance Gene Identifier-predicted resistance of *H. pylori* isolates.

*H. pylori* Strain	RGI Criteria	ARO Term	SNP	Detection Criteria	% Identity of Matching Region	% Length of Reference Sequence	Drug Class	Resistance Mechanism	AMR Gene Family	Antibiotic	AST Source
004	Strict	*vanT* in *vanG* cluster	NA	Protein homolog model	32.26	52.95	Glycopeptide antibiotic	Antibiotic target alteration	Glycopeptide resistance gene cluster; vanT	Vancomycin	-
	Strict	*Helicobacter pylori* *frxA* mutation conferring resistance to metronidazole	A16T, Y62D	Protein variant model	96.77	100	Nitroimidazole antibiotic	Antibiotic target alteration	Antibiotic-resistant *Helicobacter pylori* nitroreductase	Metronidazole	Curated-R
	Strict	*Helicobacter pylori* *pbp2* mutants conferring resistance to amoxicillin	S494H, E572G	Protein variant model	98.13	100	Cephalosporin; penicillin beta-lactam	Antibiotic target alteration	Penicillin-binding protein mutations conferring resistance to beta-lactam antibiotics	Amoxicillin	Curated-R
	Strict	*Helicobacter pylori* *rpoB* mutation conferring resistance to rifampicin	I837V	Protein variant model	99.1	100	Fluoroquinolone antibiotic; rifamycin antibiotic	Antibiotic target alteration; antibiotic target replacement	Rifamycin-resistant beta-subunit of RNA polymerase (rpoB)	Levofloxacin; rifampin	Curated-R
	Strict	*Helicobacter pylori* *rdxA* mutation conferring resistance to metronidazole	T31E, C49T, D59N	Protein variant model	95.24	100	Nitroimidazole antibiotic	Antibiotic target alteration	Antibiotic-resistant *Helicobacter pylori* nitroreductase	Metronidazole	Curated-R
	Strict	*Helicobacter pylori* 23S rRNA with mutation conferring resistance to clarithromycin	c1707t, a2144g	rRNA gene variant model	99.19	99.93	Macrolide antibiotic	Antibiotic target alteration	23S rRNA with mutation conferring resistance to macrolide antibiotics	Clarithromycin	Curated-R
117	Strict	*vanTr* gene in *vanL* cluster	NA	Protein homolog model	36	103.01	Glycopeptide antibiotic	antibiotic target alteration	glycopeptide resistance gene cluster; vanT	vancomycin	-
	Strict	*Helicobacter pylori* *pbp2* mutants conferring resistance to amoxicillin	S494H, E572G	Protein variant model	98.13	100.51	Cephalosporin; penicillin beta-lactam	antibiotic target alteration	Penicillin-binding protein mutations conferring resistance to beta-lactam antibiotics	amoxicillin	Curated-R
	Strict	*Helicobacter pylori* *pbp3* conferring resistance to amoxicillin	D2N	Protein variant model	95.93	100	Cephalosporin; penicillin beta-lactam	Antibiotic target alteration	Penicillin-binding protein mutations conferring resistance to beta-lactam antibiotics	Amoxicillin	Curated-R
	Strict	*Helicobacter pylori* *rdxA* mutation conferring resistance to metronidazole	T31E, C49T, D59N	Protein variant model	94.29	100	Nitroimidazole antibiotic	Antibiotic target alteration	Antibiotic-resistant *Helicobacter pylori* nitroreductase	Metronidazole	Curated-R
	Strict	*Helicobacter pylori* *frxA* mutation conferring resistance to metronidazole	Y62D	Protein variant model	100	100	Nitroimidazole antibiotic	Antibiotic target alteration	Antibiotic-resistant *Helicobacter pylori* nitroreductase	Metronidazole	Curated-R
	Strict	*Helicobacter pylori* 23S rRNA with mutation conferring resistance to clarithromycin	c1707t, a2144g	rRNA gene variant model	99.54	94.45	Macrolide antibiotic	Antibiotic target alteration	23S rRNA with mutation conferring resistance to macrolide antibiotics	Clarithromycin	Curated-R
189	Strict	*vanT* in *vanG* cluster	NA	Protein homolog model	32.88	51.69	Glycopeptide antibiotic	Antibiotic target alteration	Glycopeptide resistance gene cluster; *vanT*	Vancomycin	-
	Strict	*Helicobacter pylori* *pbp2* mutants conferring resistance to amoxicillin	S494H, E572G	Protein variant model	98.13	100	Cephalosporin; penicillin beta-lactam	Antibiotic target alteration	Penicillin-binding protein mutations conferring resistance to beta-lactam antibiotics	Amoxicillin	Curated-R
	Strict	*Helicobacter pylori* *rdxA* mutation conferring resistance to metronidazole	T31E, H97T, P106S, C49T, D59N, K64N	Protein variant model	95.71	100	nitroimidazole antibiotic	Antibiotic target alteration	Antibiotic-resistant *Helicobacter pylori* nitroreductase	Metronidazole	Curated-R
	Strict	*Helicobacter pylori* 23S rRNA with mutation conferring resistance to clarithromycin	c1707t, a2144g	rRNA gene variant model	99.19	99.97	macrolide antibiotic	antibiotic target alteration	23S rRNA with mutation conferring resistance to macrolide antibiotics	Clarithromycin	Curated-R

NA: ‘not available’ (no data found).

## Data Availability

The data supporting the study findings are included in the article and Supplementary Materials.

## Supplementary Materials

The following supporting information can be downloaded at: https://www.mdpi.com/article/10.3390/antibiotics14090944/s1. Table S1: Virulome profiling of *H. pylori* clinical isolates from Southern Thailand.

## Abbreviations

The following abbreviations are used in this manuscript:

AMR	antimicrobial resistance
MIC	minimal inhibitory concentration
MALDI-TOF MS	matrix-assisted laser desorption ionisation-time-of-flight mass spectrometry
