# Supplementary material for: Whole-Genome Sequencing and Antibiotic Resistance Profiling of Helicobacter pylori Isolates from a Tertiary Hospital in Southern Thailand
Source: Antibiotics (Basel). 2025 Sep 18;14(9):944. doi: 10.3390/antibiotics14090944 (PMC12466863; doi:10.3390/antibiotics14090944)
Supplement: Supplementary file 1 [file antibiotics-14-00944-s001.zip › antibiotics-3808062-supplementary.pdf]

Table S1. Virulome profiling of *H. pylori* clinical isolates from Southern Thailand.

GENE	ACCESSION	DESCRIPTION		%IDENTITY		
			**Total number of VFs**	**115**	**116**	**118**
			**FUNCTION**	**H. pylori 004**	**H. pylori 117**	**H. pylori 189**
babA/hopS	WP_000716249	(babA/hopS) outer membrane protein adhesin	BabA (VF0053) - Adherence (VFC0001)	-	89.16	90.78
babB/hopT	WP_000753173	(babB/hopT) outer membrane protein adhesin	BabA (VF0053) - Adherence (VFC0001)	-	90.93	92.33
cag1	WP_000785752	(cag1) type IV secretion system protein Cag1	Type IV secretion system (VF0060) - Effector delivery system (VFC0086)	97.41	97.13	96.84
cag2		(cag2) type IV secretion system protein Cag2	Type IV secretion system (VF0060) - Effector delivery system (VFC0086)	-	-	92.1
cag3	WP_000488766	(cag3) type IV secretion system protein Cag3 component outer membrane subcomplex	Type IV secretion system (VF0060) - Effector delivery system (VFC0086)	94.33	95.15	94.61
cagA	WP_000180747	(cagA) cag T4SS translocated effector CagA	CagA (VF0059) - Effector delivery system (VFC0086)	94.83	93.36	94.35
cagD	WP_000609477	(cagD) type IV secretion system protein Cag24/CagD	Type IV secretion system (VF0060) - Effector delivery system (VFC0086)	94.71	95.35	95.83
cagF	WP_000814259	(cagF) type IV secretion system protein Cag22/CagF chaperone-like protein for CagA	Type IV secretion system (VF0060) - Effector delivery system (VFC0086)	96.9	96.53	95.54
cagG	WP_000855900	(cagG) type IV secretion system protein Cag21/CagG	Type IV secretion system (VF0060) - Effector delivery system (VFC0086)	96.97	96.74	97.67

GENE	ACCESSION	DESCRIPTION		%IDENTITY		
			**Total number of VFs**	**115**	**116**	**118**
			**FUNCTION**	**H. pylori 004**	**H. pylori 117**	**H. pylori 189**
cagH	WP_010875497	(cagH) type IV secretion system protein Cag20/CagH	Type IV secretion system (VF0060) - Effector delivery system (VFC0086)	97.66	97.75	96.77
cagI	WP_000649592	(cagI) type IV secretion system protein Cag19/CagI	Type IV secretion system (VF0060) - Effector delivery system (VFC0086)	95.9	95.81	95.72
cagM	WP_000879904	(cagM) type IV secretion system protein Cag16/CagM transmembrane channel protein	Type IV secretion system (VF0060) - Effector delivery system (VFC0086)	98.67	97.44	98.14
cagN	WP_000835501	(cagN) type IV secretion system protein Cag17/CagN membrane-associated protein	Type IV secretion system (VF0060) - Effector delivery system (VFC0086)	95.77	96.96	98.15
cagP	WP_001863356	(cagP) type IV secretion system protein Cag15/CagP	Type IV secretion system (VF0060) - Effector delivery system (VFC0086)	96.05	96.19	95.02
cagQ	WP_000938081	(cagQ) type IV secretion system protein Cag14/CagQ	Type IV secretion system (VF0060) - Effector delivery system (VFC0086)	98.16	88.42	89.42
cagS	WP_000069596	(cagS) type IV secretion system protein Cag13/CagS	Type IV secretion system (VF0060) - Effector delivery system (VFC0086)	97.12	97.97	97.8
cagU	WP_001000423	(cagU) type IV secretion system protein Cag11/CagU	Type IV secretion system (VF0060) - Effector delivery system (VFC0086)	96.5	96.58	96.11
cagZ	WP_000418809	(cagZ) type IV secretion system protein Cag6/CagZ	Type IV secretion system (VF0060) - Effector delivery system (VFC0086)	97.83	97.67	98.67

GENE	ACCESSION	DESCRIPTION		%IDENTITY		
			Total number of VFs	115	116	118
			FUNCTION	H. pylori 004	H. pylori 117	H. pylori 189
cds6	WP_000757130	(cds6) LD-carboxypeptidase	Flagella (VF0051) - Motility (VFC0204)	95.67	95.16	94.46
cheA	WP_000342347	(cheA) histidine kinase CheA	Flagella (VF0051) - Motility (VFC0204)	95.05	94.92	95.62
cheV1	WP_000785454	(cheV1) chemotaxis coupling protein CheV1	Flagella (VF0051) - Motility (VFC0204)	96.69	96.27	97.41
cheV2	WP_000818647	(cheV2) chemotaxis coupling protein CheV2	Flagella (VF0051) - Motility (VFC0204)	95.62	97.22	96.59
cheV3	WP_000467802	(cheV3) chemotaxis coupling protein CheV3	Flagella (VF0051) - Motility (VFC0204)	96.01	95.47	96.24
cheW	WP_000070768	(cheW) chemotaxis coupling protein CheW	Flagella (VF0051) - Motility (VFC0204)	94.57	94.78	94.37
cheY	WP_000772151	(cheY) chemotaxis response regulator CheY	Flagella (VF0051) - Motility (VFC0204)	94.4	94.4	97.33
flaA	WP_000885496	(flaA) flagellin A FlaA	Flagella (VF0051) - Motility (VFC0204)	97.39	96.22	97.13
flaB	WP_000010001	(flaB) flagellin B FlaB	Flagella (VF0051) - Motility (VFC0204)	96.5	96.7	96.83
flaG	WP_000245963	(flaG) a negative regulator of flagellar assembly	Flagella (VF0051) - Motility (VFC0204)	97.78	95.28	95.28
flgA	WP_000697639	(flgA) flagellar basal body P-ring biosynthesis protein FlgA	Flagella (VF0051) - Motility (VFC0204)	92.24	93.76	96.19
flgB		(flgB) flagellar basal body rod protein FlgB	Flagella (VF0051) - Motility (VFC0204)	95.39	96.51	97.6
flgC	WP_000480097	(flgC) flagellar basal-body rod protein FlgC	Flagella (VF0051) - Motility (VFC0204)	96.09	96.09	96.09

GENE	ACCESSION	DESCRIPTION		%IDENTITY		
			Total number of VFs	115	116	118
			FUNCTION	H. pylori 004	H. pylori 117	H. pylori 189
flgD	WP_000963306	(flgD) flagellar basal-body rod modification protein FlgD	Flagella (VF0051) - Motility (VFC0204)	94.7	93.55	96.76
flgE	WP_001000225	(flgE) flagellar hook protein	Flagella (VF0051) - Motility (VFC0204)	96.48	96.53	96.42
flgE_1	WP_000946389	(flgE_1) flagellar hook protein	Flagella (VF0051) - Motility (VFC0204)	97.45	97.36	97.96
flgG	WP_001179202	(flgG) flagellar basal-body rod protein FlgG	Flagella (VF0051) - Motility (VFC0204)	95.56	96.3	96.67
flgG_2	WP_000946438	(flgG_2) flagellar basal-body rod protein (flgG)	Flagella (VF0051) - Motility (VFC0204)	94.17	97.08	96.07
flgH	WP_000709989	(flgH) flagellar L-ring protein precursor FlgH	Flagella (VF0051) - Motility (VFC0204)	96.64	96.92	97.48
flgI	WP_000832067	(flgI) flagellar P-ring protein precursor FlgI	Flagella (VF0051) - Motility (VFC0204)	96.5	97.47	96.31
flgK	WP_000508899	(flgK) flagellar hook-associated protein 1 FlgK	Flagella (VF0051) - Motility (VFC0204)	96.32	96.92	96.92
flgL	WP_001266636	(flgL) flagellar hook-associated protein 3 FlgL	Flagella (VF0051) - Motility (VFC0204)	95.45	94.41	95.37
flgM	WP_001863090	(flgM) negative regulator of flagellin synthesis	Flagella (VF0051) - Motility (VFC0204)	97.06	92.61	93.63
flgR	WP_000684509	(flgR) response regulator	Flagella (VF0051) - Motility (VFC0204)	96.42	96.25	96.68
flgS	WP_000748581	(flgS) signal-transducing protein histidine kinase	Flagella (VF0051) - Motility (VFC0204)	96.07	96.51	98.34
flhA	WP_001262893	(flhA) flagellar biosynthesis protein	Flagella (VF0051) - Motility (VFC0204)	96.46	97.18	97.46
flhB	WP_000796857	(flhB) flagellar biosynthesis protein	Flagella (VF0051) - Motility (VFC0204)	95.82	96.38	96.38

GENE	ACCESSION	DESCRIPTION		%IDENTITY		
			**Total number of VFs**	**115**	**116**	**118**
			**FUNCTION**	**H. pylori 004**	**H. pylori 117**	**H. pylori 189**
flhB2	WP_001044050	(flhB2) ABC transporter putative	Flagella (VF0051) - Motility (VFC0204)	95.97	97.07	97.44
flhF	WP_001862801	(flhF) flagellar biosynthesis protein	Flagella (VF0051) - Motility (VFC0204)	97.68	97.53	97.46
fliA	WP_000602430	(fliA) flagellar biosynthesis sigma factor FliA	Flagella (VF0051) - Motility (VFC0204)	96.01	95.24	96.61
fliD	WP_010875540	(fliD) flagellar capping protein FliD	Flagella (VF0051) - Motility (VFC0204)	95.16	96.15	96.15
fliE	WP_001147918	(fliE) flagellar hook-basal body complex protein FliE	Flagella (VF0051) - Motility (VFC0204)	96.36	95.14	95.76
fliF	WP_000364750	(fliF) flagellar M-ring protein FliF	Flagella (VF0051) - Motility (VFC0204)	96.6	96.42	97.36
fliG	WP_000201853	(fliG) flagellar motor switch protein G	Flagella (VF0051) - Motility (VFC0204)	97.29	96.22	97.87
fliH	WP_000056222	(fliH) flagellar assembly protein H	Flagella (VF0051) - Motility (VFC0204)	97.94	96.65	96.53
fliI	WP_001128709	(fliI) flagellum-specific ATP synthase FliI	Flagella (VF0051) - Motility (VFC0204)	97.17	97.24	96.78
fliL	WP_000797017	(fliL) flagellar basal body protein FliL	Flagella (VF0051) - Motility (VFC0204)	96.74	97.28	96.19
fliM	WP_000763575	(fliM) flagellar motor switch protein FliM	Flagella (VF0051) - Motility (VFC0204)	97.65	96.9	97.18
fliN	WP_000004827	(fliN) flagellar motor switch protein FliN	Flagella (VF0051) - Motility (VFC0204)	97.04	96.5	97.04
fliP		(fliP) flagellar biosynthetic protein FliP	Flagella (VF0051) - Motility (VFC0204)	97.85	97.46	97.46
fliQ	WP_000445741	(fliQ) flagellar biosynthesis protein	Flagella (VF0051) - Motility (VFC0204)	95.86	95.51	95.51

GENE	ACCESSION	DESCRIPTION		%IDENTITY		
			Total number of VFs	115	116	118
			FUNCTION	H. pylori 004	H. pylori 117	H. pylori 189
fliR	WP_000883720	(fliR) flagellar biosynthetic protein FliR	Flagella (VF0051) - Motility (VFC0204)	95.83	96.48	98.31
fliS	WP_001199081	(fliS) flagellar protein FliS	Flagella (VF0051) - Motility (VFC0204)	97.61	96.57	97.89
fliY	WP_001150629	(fliY) flagellar motor switch protein	Flagella (VF0051) - Motility (VFC0204)	95.49	94.39	95.19
futB	WP_000487430	(futB) fucosyltransferase	Lewis antigen (VF0057) - Immune modulation (VFC0258)	86.74	-	89.02
futC1	WP_010875440	(futC1) alpha-(12)-fucosyltransferase	Lewis antigen (VF0057) - Immune modulation (VFC0258)	96.6	96.6	97.28
futC2	WP_000874844	(futC2) alpha-(12)-fucosyltransferase	Lewis antigen (VF0057) - Immune modulation (VFC0258)	94.46	92.73	95.86
gluE	WP_001186109	(gluE) UDP-glucose 4-epimerase	LPS (VF0056) - Immune modulation (VFC0258)	96.33	95.94	96.62
gluP	WP_001174195	(gluP) glucose/galactose transporter	LPS (VF0056) - Immune modulation (VFC0258)	97.22	96.32	96.89
hopZ	WP_042960771	(hopZ) outer membrane protein adhesin	HopZ (VF0054) - Adherence (VFC0001)	90.39	-	-
HP_RS02435	WP_000442290	(HP_RS02435) neuraminyllactose-binding hemagglutinin	Flagella (VF0051) - Motility (VFC0204)	95.14	95.02	97.25
HP_RS03030	WP_000251148	(HP_RS03030) chemotaxis protein	Flagella (VF0051) - Motility (VFC0204)	97.24	95.33	94.8
HP_RS03480	WP_000996418	(HP_RS03480) RNA polymerase factor sigma-54	Flagella (VF0051) - Motility (VFC0204)	95.74	95.5	95.66
HP_RS04690	WP_001091835	(HP_RS04690) zinc ribbon domain-containing protein	Flagella (VF0051) - Motility (VFC0204)	95.03	95.56	96.86
HP_RS07005	WP_000380989	(HP_RS07005) alpha-12/4 Glc transferase	LPS (VF0056) - Immune modulation (VFC0258)	91.39	92.57	93.35

GENE	ACCESSION	DESCRIPTION		%IDENTITY		
			Total number of VFs	115	116	118
			FUNCTION	H. pylori 004	H. pylori 117	H. pylori 189
HP_RS07240	WP_000603368	(HP_RS07240) META domain-containing protein	Flagella (VF0051) - Motility (VFC0204)	96.26	97.11	96.26
HP0256	WP_000712783	(HP0256) involved in motility and cell envelope architecture	Flagella (VF0051) - Motility (VFC0204)	95.8	95.34	95.57
hpaA2	WP_000715711	(hpaA2) ahesin protein	Flagella (VF0051) - Motility (VFC0204)	88.52	88.12	94.67
kdtB	WP_001169234	(kdtB) lipopolysaccharide core biosynthesis protein	LPS (VF0056) - Immune modulation (VFC0258)	97.47	94.73	96.2
lpxB	WP_001142178	(lpxB) lipid A disaccharide synthetase	LPS (VF0056) - Immune modulation (VFC0258)	96.12	96.03	95.75
motA	WP_000366185	(motA) flagellar motor protein	Flagella (VF0051) - Motility (VFC0204)	97.29	96.51	97.16
motB	WP_001085308	(motB) flagellar motor protein	Flagella (VF0051) - Motility (VFC0204)	96.72	96	95.48
napA	WP_000846461	(napA) neutrophil activating protein NapA	HP-NAP (VF0052) - Immune modulation (VFC0258)	94.48	96.55	97.01
oipA/hopH	WP_000709741	(oipA/hopH) outer inflammatory protein A adhesin	OipA (VF0266) - Immune modulation (VFC0258)	93.83	94.63	95.56
pdxA	WP_001075036	(pdxA) 4-hydroxythreonine-4-phosphate dehydrogenase	Flagella (VF0051) - Motility (VFC0204)	93.94	94.59	95.89
pdxJ	WP_001210849	(pdxJ) pyridoxine 5'-phosphate synthase	Flagella (VF0051) - Motility (VFC0204)	95.82	95.06	95.31
pflA	WP_000266337	(pflA) paralysed flagella protein (pflA)	Flagella (VF0051) - Motility (VFC0204)	94.88	95.9	95.89
pseB	WP_001863409	(pseB) UDP-N-acetylglucosamine 46-dehydratase	Flagellar glycosylation/Pse biosynthetic pathway (VF0608) - Motility (VFC0204)	95.12	94.11	94.92

GENE	ACCESSION	DESCRIPTION		%IDENTITY		
			**Total number of VFs**	**115**	**116**	**118**
			**FUNCTION**	**H. pylori 004**	**H. pylori 117**	**H. pylori 189**
pseC	WP_000657303	(pseC) flagellar modification protein aminotransferase PseC	Flagellar glycosylation/Pse biosynthetic pathway (VF0608) - Motility (VFC0204)	95.29	95.57	95.65
pseFG	WP_001201399	(pseFG) CMP-N-acetylneuraminic acid synthetase	Flagellar glycosylation/Pse biosynthetic pathway (VF0608) - Motility (VFC0204)	94.66	93.56	94.08
pseH/flmH/flaG1	WP_000742697	(pseH/flmH/flaG1) UDP-4-amino-46-dideoxy-N-acetyl-beta-L-altrosamine N-acetyltransferase; Pseudaminic acid biosynthesis protein H	Flagellar glycosylation/Pse biosynthetic pathway (VF0608) - Motility (VFC0204)	94.66	93.74	95.58
pseI	WP_000941291	(pseI) pseudaminic acid synthase	Flagellar glycosylation/Pse biosynthetic pathway (VF0608) - Motility (VFC0204)	94.53	94.92	95.6
rfaC	WP_000684682	(rfaC) lipopolysaccharide heptosyltransferase-1	LPS (VF0056) - Immune modulation (VFC0258)	91.69	91.98	92.57
rfaJ	WP_000022419	(rfaJ) alpha-16 Glc transferase	LPS (VF0056) - Immune modulation (VFC0258)	91.96	92.31	92.76
rfbD	WP_000659810	(rfbD) GDP-D-mannose dehydratase	LPS (VF0056) - Immune modulation (VFC0258)	95.9	96.6	96.77
rfbM	WP_000694809	(rfbM) mannose-6-phosphate isomerase	LPS (VF0056) - Immune modulation (VFC0258)	95.12	93.45	94.55
sabA/hopP	WP_000542820	(sabA/hopP) outer membrane protein adhesin	SabA (VF0055) - Adherence (VFC0001)	91.47	90.95	
sabB/hopO	WP_000542818	(sabB/hopO) outer membrane protein adhesin	SabA (VF0055) - Adherence (VFC0001)			87.56

GENE	ACCESSION	DESCRIPTION		%IDENTITY		
			Total number of VFs	115	116	118
			FUNCTION	H. pylori 004	H. pylori 117	H. pylori 189
tlpA	WP_000033553	(tlpA) membrane-bound chemoreceptor sensing arginine and bicarbonate	Flagella (VF0051) - Motility (VFC0204)	96.06	95.51	96.8
tlpB	WP_000971620	(tlpB) membrane-bound chemoreceptor sensing pH and autoinducer-2	Flagella (VF0051) - Motility (VFC0204)	95.29	95.41	96.11
tlpC	WP_000843374	(tlpC) membrane-bound chemoreceptor	Flagella (VF0051) - Motility (VFC0204)	96.09	96.83	96.09
ureA	WP_000779223	(ureA) urease alpha subunit UreA	Urease (VF0050) - Stress survival (VFC0282)	97.63	97.63	98.88
ureB	WP_000724295	(ureB) urease beta subunit UreB urea amidohydrolase	Urease (VF0050) - Stress survival (VFC0282)	96.84	96.96	97.48
ureE	WP_000583097	(ureE) urease accessory protein (ureE) metallochaperone	Urease (VF0050) - Stress survival (VFC0282)	97.08	95.71	96.69
ureF	WP_000357404	(ureF) urease accessory protein (ureF)	Urease (VF0050) - Stress survival (VFC0282)	96.6	96.47	97.52
ureG	WP_000238762	(ureG) urease accessory protein (ureG)	Urease (VF0050) - Stress survival (VFC0282)	97.5	97.33	98.67
ureH	WP_001099471	(ureH) urease accessory protein (ureH)	Urease (VF0050) - Stress survival (VFC0282)	96.36	94.18	95.85
ureI	WP_000901247	(ureI) acid-activated urea channel protein	Urease (VF0050) - Stress survival (VFC0282)	97.79	96.6	97.11
vacA	WP_000405496	(vacA) vacuolating cytotoxin	VacA (VF0058) - Exotoxin (VFC0235)	-	91.34	93.55
virB1/cag4	WP_000462193	(virB1/cag4) type IV secretion system protein Cag4 VirB1 homolog	Type IV secretion system (VF0060) - Effector delivery system (VFC0086)	89.41	90.2	92.16

GENE	ACCESSION	DESCRIPTION		%IDENTITY		
			**Total number of VFs**	**115**	**116**	**118**
			**FUNCTION**	**H. pylori 004**	**H. pylori 117**	**H. pylori 189**
virB11	WP_000133885	(virB11) type IV secretion system ATPase VirB11 homolog	Type IV secretion system (VF0060) - Effector delivery system (VFC0086)	97.48	97.89	97.89
virB2/cagC	WP_000668162	(virB2/cagC) type IV secretion system protein Cag25/CagC VirB2 homolog	Type IV secretion system (VF0060) - Effector delivery system (VFC0086)	94.54	95.69	95.4
virB4/cagE	WP_000495985	(virB4/cagE) type IV secretion system protein Cag23/CagE VirB4 homolog	Type IV secretion system (VF0060) - Effector delivery system (VFC0086)	97.56	97.02	97.46
virB5/cagL	WP_000855299	(virB5/cagL) type IV secretion system protein Cag18/CagL VirB5 homolog	Type IV secretion system (VF0060) - Effector delivery system (VFC0086)	96.64	95.24	97.2
virB6/cagW	WP_000481884	(virB6/cagW) type IV secretion system protein Cag9/CagW VirB6 homolog	Type IV secretion system (VF0060) - Effector delivery system (VFC0086)	97.76	97.08	96.7
virB7/cagT	WP_000776464	(virB7/cagT) type IV secretion system protein Cag12/CagT VirB7 homolog	Type IV secretion system (VF0060) - Effector delivery system (VFC0086)	97.98	97.86	98.34
virB8/cagV	WP_000900785	(virB8/cagV) type IV secretion system protein Cag10/CagV VirB8 homolog	Type IV secretion system (VF0060) - Effector delivery system (VFC0086)	98.16	97.63	97.1
virB9/cagX	WP_000529349	(virB9/cagX) type IV secretion system protein Cag8/CagX VirB9 homolog	Type IV secretion system (VF0060) - Effector delivery system (VFC0086)	96.94	97.51	97.9
virD4/cag5	WP_000389328	(virD4/cag5) type IV secretion system protein Cag5 VirD4 homolog	Type IV secretion system (VF0060) - Effector delivery system (VFC0086)	95.24	95.55	95.5
wbcJ	WP_001002442	(wbcJ) GDP fucose synthase	LPS (VF0056) - Immune modulation (VFC0258)	95.61	96.25	96.78

GENE	ACCESSION	DESCRIPTION		%IDENTITY		
			**Total number of VFs**	**115**	**116**	**118**
			**FUNCTION**	**H. pylori 004**	**H. pylori 117**	**H. pylori 189**
wbpB		(wbpB) lipopolysaccharide biosynthesis protein	LPS (VF0056) - Immune modulation (VFC0258)	95.68	95.26	95.36
ylxH	WP_001064464	(ylxH) ATP-binding protein	Flagella (VF0051) - Motility (VFC0204)	97.06	95.03	96.5
